# PET imaging of tissue reactions in the implanted cochlea: results of a pilot study

**DOI:** 10.3389/fimmu.2025.1601742

**Published:** 2025-07-10

**Authors:** Philipp Deutsch, Jens P. Bankstahl, Martin Mamach, Michael Willmann, Frank M. Bengel, Thomas Lenarz, Georg Berding, Verena Scheper, Jana Schwieger

**Affiliations:** ^1^ Department of Nuclear Medicine, Hannover Medical School, Hannover, Germany; ^2^ Cluster of Excellence Hearing4all, Hannover Medical School and University of Oldenburg, Hannover, Germany; ^3^ Department of Radiation Protection and Medical Physics, Hannover Medical School, Hannover, Germany; ^4^ Department of Otorhinolaryngology, Hannover Medical School, Hannover, Germany; ^5^ NIFE - Lower Saxony Center for Biomedical Engineering, Implant Research and Development, Hannover, Germany

**Keywords:** cochlear implantation, tissue growth, inflammation, fibrosis, guinea pig, positron emission tomography/computed tomography

## Abstract

**Purpose:**

After cochlear implantation, molecular processes at the electrode–nerve interface significantly influence the variability in clinical outcomes. The present study investigates molecular processes in a guinea pig model of cochlear implant (CI) using positron emission tomography/computed tomography (PET/CT) and correlates the imaging findings with histological analyses.

**Methods:**

Animals were examined with PET in the 3 weeks and 9–12 months post-implantation using the inflammation marker [^18^F]FDG and, at the later time points, [^68^Ga]FAPI-46 as a marker for fibrosis. Tracer accumulation in the cochlea was determined from PET imaging based on the co-registered CT. Nine animals (seven with unilateral CI) were included. Uptake in non-implanted cochleae served as reference. Tissue growth around the implant was evaluated histologically.

**Results:**

Post-implantation, [^18^F]FDG uptake was significantly increased when pooling early and late in investigation time points, while after 1 year, [^68^Ga]FAPI-46 uptake was increased inside the cochlear. Cochlear volumes measured by CT did not show significant differences between compared groups. Tissue growth around the implant was observed in all animals, with a trend toward increased growth associated with insertion depth. However, no clear correlation was observed between the extent of tissue growth and the uptake intensities of FDG and FAPI.

**Discussion:**

The data indicate that increased accumulation of PET biomarkers in the cochlea after implantation can be detected in guinea pigs using a dedicated PET/CT. Given the high resolution of current clinical PET/CT devices, this method is expected to be suitable for use in patients, particularly for assessing the effect of anti-inflammatory or anti-fibrotic therapies.

## Introduction

In most cases, cochlear implantation is currently the only option to treat sensorineural hearing loss and enable speech perception (1). However, the outcome of cochlear implantation varies between patients and not all benefit from the treatment to the same extent. Approximately a quarter of patients still have word or sentence comprehension of less than 30% 1 year after implantation (2), or function declines over time, leading to a loss of speech perception. Additionally, in patients with residual hearing who are provided with a cochlear implant (CI), the success of preserving residual hearing varies and so do the benefits of electroacoustic stimulation. The causes of poor outcomes post-implantation are diverse and include biological and audiological factors, hearing loss history, electrode positioning, cognitive factors, and tissue responses around the implant as a result of insertion trauma and the foreign body reaction (3). The underlying mechanisms involved in the tissue responses to a CI are not yet fully understood due to the poor accessibility of the cochlea, which makes observation of ongoing tissue reactions almost impossible. Previous findings of cellular and molecular mechanisms were primarily based on and limited to histological evaluations post mortem, from both animal experiments and CI patients. In addition to direct tissue damage caused by insertion trauma, both acute and chronic inflammatory responses can occur, leading to cell death and the subsequent formation of a fibrous, and later bony, encapsulation around the electrode (4). This lowers the amount of functional tissue and hinders electrical signal transmission, which is partially observable by loss of hearing function and an increase in impedance (5).

There are efforts to establish computed tomography (CT) as a non-invasive imaging method of the cochlea in animal studies and patients to detect and quantify tissue growth around the electrode; however, this is mainly limited to bony tissue and requires high resolution and radiation exposure (6, 7). Thus, there is an urgent need for diagnostic methods that can non-invasively monitor tissue responses to a CI in order to decipher its impact on CI performance, determine the need for therapeutic intervention, and monitor therapeutic efficacy. In preclinical experiments, the ability to longitudinally image implanted animals reduces the number of animals needed, and in patients, such technology could investigate whether specific tissue reactions correlate with specific differences in CI performance. Imaging may reveal differences in tissue responses to relatively unspecific implantation-related treatments, like prophylactic administration of glucocorticoids during implantation, compared to personalized therapy over the entire period of implantation. Therefore, establishing a non-invasive method that can visualize tissue reactions in the cochlea would be a great benefit.

Positron emission tomography combined with computed tomography (PET/CT) and the use of specific radioactive markers are non-invasive nuclear medicine investigation methods and have the potential to address this need by measuring underlying molecular processes in the cochlea. For example, [^18^F]FDG (fluorine-18-fluorodeoxyglucose, FDG) PET has been a proven method for decades to detect inflammation, especially in the area of implants (8). In recent years, radiopharmaceuticals have been developed to visualize activated fibroblasts, such as [^68^Ga]FAPI-46 (gallium-68-fibroblast activation protein inhibitor-46, FAPI), which binds as an inhibitor to the fibroblast activation protein alpha (9, 10). This tracer was originally developed for the detection of tumors via their connective tissue. While its application has been extended to non-malignant diseases associated with fibrosis, it has not yet been used in connection with CIs. The successful use of these tracers for the diagnosis of implantation-related tissue reactions requires that they enter the cochlea and that the resolution of the PET allows the detection of the radiopharmaceuticals in the relatively small cochlea. A prerequisite for marker accumulation in the cochlea is the passage of the blood–labyrinth barrier. This includes, in particular, the endothelial cells of the stria vascularis (11, 12). Since no data are available in this regard, perhaps the permeability of a radiopharmaceutical across the blood–brain barrier can provide a first clue. This is undoubtedly the case for FDG, as it is well established in the diagnosis of cerebral energy metabolism (13). For FAPI, experimental biodistribution studies indicate some uptake in the brain (14). Furthermore, an increase in permeability of the blood–labyrinth barrier is to be expected due to inflammatory processes as a result of implantation (15, 16). In this respect, both radiopharmaceuticals appear promising—FDG, for detecting acute inflammatory response accompanied by a massive (immune) cell reactivity, and FAPI, for the later tissue response with (fibrotic) encapsulation of the implant.

A very common animal model in hearing and CI research is the guinea pig. The dimensions of the guinea pig cochlea (diameter at the base, height) are approximately 3–5 mm (17, 18). The spatial resolution of state-of-the-art PET devices for preclinical imaging is approximately 1.4 mm (full width at half maximum) (19, 20). Thus, the size of the guinea pig cochlea corresponds approximately to two to three times the resolution (full width at half maximum) and an 80% recovery of the signal from the cochlea can be expected (20, 21). From a methodological point of view, this makes the guinea pig a suitable model for a pilot study on PET imaging after CI implantation.

The present work is a pilot study reporting on the feasibility of imaging molecular processes in the cochlea in a model of traumatic electrode insertion in guinea pigs (5). The aims were to detect early and late inflammatory changes post-implantation, to visualize fibrotic activity at the later stage, and to correlate these findings with histology and electrode position as determined by µCT.

## Materials and methods

The study started as an exploratory case study to assess the potential of PET imaging as a monitoring tool after CI implantation. Retrospective case grouping revealed tendencies regarding the development of inflammation and fibrosis after implantation. While we acknowledge that this work is not a substitute for a more comprehensive study, the findings provide a valuable foundation for designing larger-scale investigations into the use of PET imaging for monitoring after CI implantation.

### Animals and experimental groups

The study included nine adult guinea pigs (male and female, Dunkin Hartley, Charles River Laboratories, France). Animals were housed in a temperature- and humidity-controlled room, exposed to a 24-h light–dark cycle (14 h/10 h) with free access to food and water. Two non-implanted animals were examined with two different radiopharmaceuticals ([^18^F]FDG and [^68^Ga]FAPI-46) to establish PET imaging. One animal (female) was examined early after unilateral CI with [^18^F]FDG, starting 1 week after implantation, followed by two additional scans on a weekly basis to observe early tissue reactions around the implant. In total, five animals were scanned with PET 1 year after implantation and another one after 9 months. Three of those animals (two male and one female) were examined with [^18^F]FDG, while the remaining three animals (female) received [^68^Ga]FAPI-46. For an overview, see **Table 1**. The average weight of animals examined during the establishment and early post-implantation phases was 438 ± 66 g, and the average weight of animals examined 1 year after implantation was 1,087 ± 95 g. All experiments were approved by the local authorities (Lower Saxony State Office for Consumer Protection and Food Safety; registration no: 20/3350) and were conducted in accordance with the German “Law on Protecting Animals” and with the European Communities Council Directive 2010/63/EU for the protection of animals used for scientific purposes.

**Table 1:** Overview of animals, implants, implantation status, time point of scanning, and radiopharmaceuticals used.

Phase of study	Animal	Scan time point after Implantation	Radiopharmaceutical	Cochlea	Implantation	Implant
Establishing scanning	GP001 ♀	n/a	[^18^F]FDG, [^68^Ga]FAPI-46	Left	No	–
				Right	No	
	GP002 ♀	n/a	[^18^F]FDG, [^68^Ga]FAPI-46	Left	No	–
				Right	No	
Scanning early post-implantation	GP003 ♀	1 week	[^18^F]FDG	Left	Yes	Thin passive electrode, 2 contacts, wire
				Right	No	
		2 weeks	[^18^F]FDG	Left	Yes	
				Right	No	
		3 weeks	[^18^F]FDG	Left	Yes	
				Right	No	
Scanning late post-implantation	GP004 ♂	1 year	[^18^F]FDG	Left	Yes	Active electrode, 5 contacts, connector
				Right	No	
	GP005 ♂	1 year	[^18^F]FDG	Left	Yes	Active electrode, 5 contacts, connector
				Right	No	
	GP006 ♀	1 year	[^18^F]FDG	Left	Yes	Active electrode, 5 contacts, connector
				Right	No	
	GP008 ♀	1 year	[^68^Ga]FAPI-46	Left	Yes	Active electrode, 4 contacts, connector
				Right	No	
	GP009 ♀	1 year	[^68^Ga]FAPI-46	Left	Yes	Active electrode, 4 contacts, connector
				Right	No	
	GP011 ♀	9 months	[^68^Ga]FAPI-46	Left	Yes	Passive electrode, wire
				Right	No	

### Anesthesia, medication regime, and surgical procedure

Surgery, CT, PET, and euthanasia were performed under general anesthesia with medetomidinhydrochloride (0.2 mg/kg), midazolam (1 mg/kg), and fentanyl (0.025 mg/kg) given intramuscularly after previous sedation with diazepam (4 mg/kg, oral). Animals received a probiotic (0.5 g, oral) 1 day before, pre- and 1 day post-anesthesia to prevent indigestion. To avoid eye desiccation, anesthetized animals received eye ointment. In addition, animals received atropine (0.5 mg/kg) or glycopyrronium bromide (0.02 mg/kg) subcutaneously to reduce bronchial secretion and salivation during anesthesia and Ringer’s solution including 5% glucose as a subcutaneous infusion (4 mL/300 g). Anesthesia was antagonized by injecting atipamezole (1 mg/kg), flumazenil (0.1 mg/kg), and naloxone (0.03 mg/kg) subcutaneously. For cochlear implantation, meloxicam (0.2 mg/kg) was given subcutaneously as an analgesic and the incision site was infiltrated with prilocaine for local anesthesia, while enrofloxacin (10 mg/kg) was given subcutaneously to prevent wound infection. Postoperative care included 3 days of oral meloxicam (0.2 mg/kg) and 7 days of oral enrofloxacin (5 mg/kg). Euthanasia was performed via intracardiac injection of pentobarbital (not less than 300 mg/kg). See **Table 2** for a summary of the administered drugs.

**Table 2:** Used drugs.

Drug	Brand	Concentration	Company	Country
Medetomidine-hydrochloride	Dormilan®	1 mg/mL	alfavet Tierarzneimittel GmbH	Germany
Midazolam	Midazolam	5 mg/mL	PANPHARMA GmbH	Germany
Fentanyl	Fentadon	50 µg/mL	Dechra Veterinary Products Deutschland GmbH	Germany
Diazepam	Diazepam-ratiopharm®	10 mg/mL	ratiopharm GmbH	Germany
Eye ointment	Bepanthen®		Bayer Vital GmbH	Germany
Prilocaine	Xylonest	1%	Aspen Germany GmbH	Germany
Probiotic	BENE-BAC® Gel		Dechra Veterinary Products Deutschland GmbH	Germany
Atropine	Atropinsulfat B. Baun	0.5 mg/mL	B. Braun Melsungen AG	Germany
Glycopyrronium bromide	Glycopyrroniumbromid Accord	200 µg/mL	Accord Healthcare B. V.	Netherlands
Ringer’s solution	Ringer-Lösung DELTAMEDICA		DELTAMEDICA GmbH	Germany
Glucose	Glucose 40% B. Braun	40%	B. Braun Melsungen AG	Germany
Atipamezole	ATIPAZOLE	5 mg/mL	Prodivet pharmaceutics	Belgium
Flumazenil	Flumazenil-hameln	0.1 mg/mL	Hameln pharma GmbH	Germany
Naloxone	Naloxon Inresa	0.4 mg/mL	Inresa Arzneimittel GmbH	Germany
Meloxicam	Metacam®	2 mg/mL	Boehringer Ingelheim Vetmedica GmbH	Germany
Enrofloxacin	Baytril®	25 mg/mL	Elanco GmbH	Germany
Meloxicam	Metacam®	0.5 mg/mL	Boehringer Ingelheimmedica GmbH	Germany
Enrofloxacin	Baytril®	2.5%	Elanco GmbH	Germany
Pentobarbital	Release®	300 mg/mL	Wirtschaftsgenossenschaft deutscher Tierärzte eG	Germany

Different types of CIs were used, which potentially differ in implantation trauma. Three of the long-term implanted animals received implants with a head connector and an electrode array with five active contacts while another two animals received an array with four active contacts. The last long-term CI animal was implanted with a dummy electrode lacking a connector and active electrodes. Advanced Bionics (AB, Sonova Holding AG, USA) provided all the electrodes. The animal implanted for 3 weeks received a passive electrode with two contacts (MEDEL, Austria). Implant assignment to the animals is given in **Table 1**.

All animals undergoing CI surgery were implanted in the left cochlea. The surgical procedure was identical for all animals and implantation was performed as previously described (22). In short, the post-auricular skin was incised and the bulla was exposed and fenestrated to open the middle ear cavity where the basal area of the cochlea was microscopically visualized. Cochleostomy was drilled using a burr of 0.6 mm diameter at a low frequency to access the scala tympani. Implantation via cochleostomy was chosen to cause more trauma (23) and, by this, to increase inflammation and fibrosis in the cochlea. Additionally, an electrode insertion trauma (EIT) was induced by repeating the insertion three times before final placement of the array or dummy. The CI was secured in place and the bulla was closed using Tetric EvoFlow^®^ (Ivoclar Vivadent, Liechtenstein). The external part of the electrode or dummy was placed under the post-auricular muscles before the muscles and skin were sutured in two layers for wound closure. For active implants, the connector was fixed on the skull with two screws and Tetric EvoFlow^®^ and the electrode was tunneled under the muscles from the connector to the bulla defect.

To avoid displacement of the electrode array due to loss of connectors over the observation time of up to 1 year (see **Table 1**), the electrode was cut outside of the bulla (lateral wall of the connector, between skin and dental cement) preventively in a second surgery after 2 months.

### Radiochemistry

#### Radiolabeling of [^68^Ga]FAPI-46

[^68^Ga]GaCl_3_ was obtained by eluting a TiO_2_-based ^68^Ge/^68^Ga radionuclide generator (GalliAd, IRE ELiT) with 1.5 mL of 0.1 M HCl, yielding 500–1,000 MBq of radioactivity. Radiolabeling of FAPI-46 was carried out by reacting (*S*)-2,2',2''-(10-(2-(4-(3-((4-((2-(2-cyano-4,4-difluoropyrrolidin-1-yl)-2-oxoethyl)carbamoyl)quinolin-6-yl)(methyl)amino)propyl)piperazin-1-yl)-2-oxoethyl)-1,4,7,10-tetraazacyclododecane-1,4,7-triyl)triacetic acid (15 nmol) in 300 µL of 1.5 M 4-(2-hydroxyethyl)-1-piperazineethanesulfonic acid (HEPES) with the freshly eluted [^68^Ga]GaCl_3_ at 100°C for 5 min. Following synthesis, the reaction mixture was diluted with 10 mL of water and passed through an activated HLB-light cartridge (Waters™, Germany). The cartridge was subsequently washed with 3 mL of water, and the purified product was eluted using 1.5 mL of ethanol. The eluate was then concentrated to approximately 100 µL under a stream of nitrogen and diluted with 0.1% Tween^®^ 80 to obtain the final formulation with an activity yield of 262 ± 126 MBq and an apparent molar activity of 17.5 ± 8.40 GBq/µmol.

#### Automated synthesis of [^18^F]FDG

[^18^F]Fluoride was produced via a (p,n) nuclear reaction using an Eclipse HP cyclotron (Siemens AG) by irradiating enriched [^18^O]H_2_O with 11-MeV protons. The synthesis of [^18^F]FDG was carried out using an automated FASTlab™ synthesizer (GE Healthcare, UK) equipped with a GMP-compliant single-use disposable cassette system. The final product was prepared for standard in-house clinical applications.

### PET/CT acquisition and reconstruction

A Siemens Inveon™ PET/CT system (Siemens Medical Solutions, USA) was used for the image acquisitions. First, an opioid-based anesthesia was induced (see above). After the onset of anesthesia, a cannula was inserted in the saphenous vein (24). The animal was supported by a heating mat to keep its physiological body temperature and placed with the head and heart region in the field of view of the PET device. After that, the radiopharmaceutical was injected. [^18^F]FDG (31.3 ± 8.4 MBq) was administered intravenously in a volume of 0.2 mL, except at 14 and 21 days after implantation, where 0.3 mL was applied. For [^68^Ga]FAPI-46, 33.8 ± 13.3 MBq was intravenously administered in a volume of 0.2 mL. Blood samples for serum glucose determination were taken before radiotracer application and after PET acquisition in the case of the [^18^F]FDG scans, in order to exclude a significantly elevated glucose level that could have affected the uptake of the radiopharmaceutical. After every 1 h of PET acquisition, a low-dose CT scan was performed with a tube voltage of 50 kV over 300 ms, and the CT images were reconstructed to a 512 × 512 × 512 image matrix (96 µm^3^ voxel size). The PET data were reconstructed iteratively (OSEM3D) with attenuation correction in 32 consecutive frames (5 × 2 s, 4 × 5 s, 3 × 10 s, 8 × 30 s, 5 × 60 s, 4 × 5 min, 3 × 10 min) to a 128 × 128 × 159 image matrix (0.78 × 0.78 × 0.80 mm).

### PET/CT data analysis

**Figure 1:**
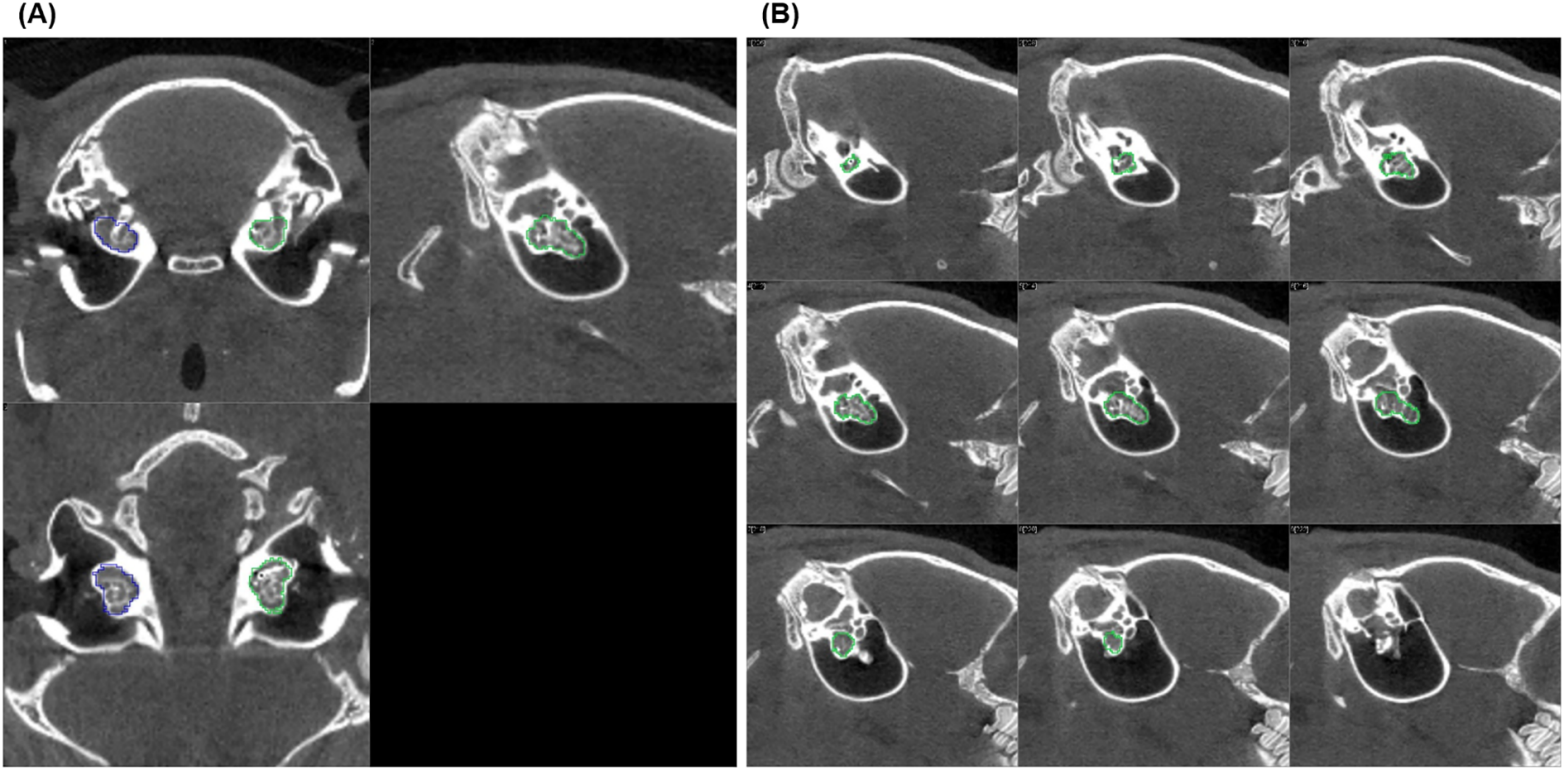
CT scan slice series of an implanted left guinea pig cochlea used to define the volume of interest (VOI): **(A)** shows three orthogonal sections (coronal, sagittal, and axial) of the implanted (green) and non-implanted (blue) cochlea, and **(B)** displays nine sagittal slices (parallel to the cochlea's central axis, every second slice is shown). The green lines drawn on each tomogram mark the outer edge of the cochlea layer by layer. Together, these lines define the VOI, which allows to read out the mean activity concentration in the cochlea after it is transferred to the inherently co-registered PET.

A mean standardized uptake value (SUVmean) was determined as a measure of the accumulation of the radiopharmaceutical in the cochlea. For this purpose, the cochlea was first visually delineated on consecutive slices in the low-dose CT inherently co-registered to the PET. In general, this was possible on approximately 17 slices (**Figure 1**). The tomogram by tomogram delineations of the cochlea were fused to a volume of interest (VOI), and the total volume (in ccm = cubic centimeter) calculated for comparison of different conditions (e.g., cochleae with and without implant). The VOIs were then transferred to the respective PET dataset to read out the mean activity concentration (in kBq/mL) in the cochlea. The latter was determined from the sequential dataset by weighting according to the recording time from consecutive frames over the total recording time. The SUVmean was then calculated from the mean activity concentrations divided by the respective injected amount of activity per animal’s body weight. A total of 13 PET examinations with 9 implanted and 17 non-implanted cochleae were analyzed as described (see **Table 1**). In addition, abnormalities possibly associated with the implantation, such as increased uptake of the radiopharmaceutical adjacent to or emanating from the cochlea, were visually assessed.

### Determination of electrode position and histological assessment

**Figure 2:**
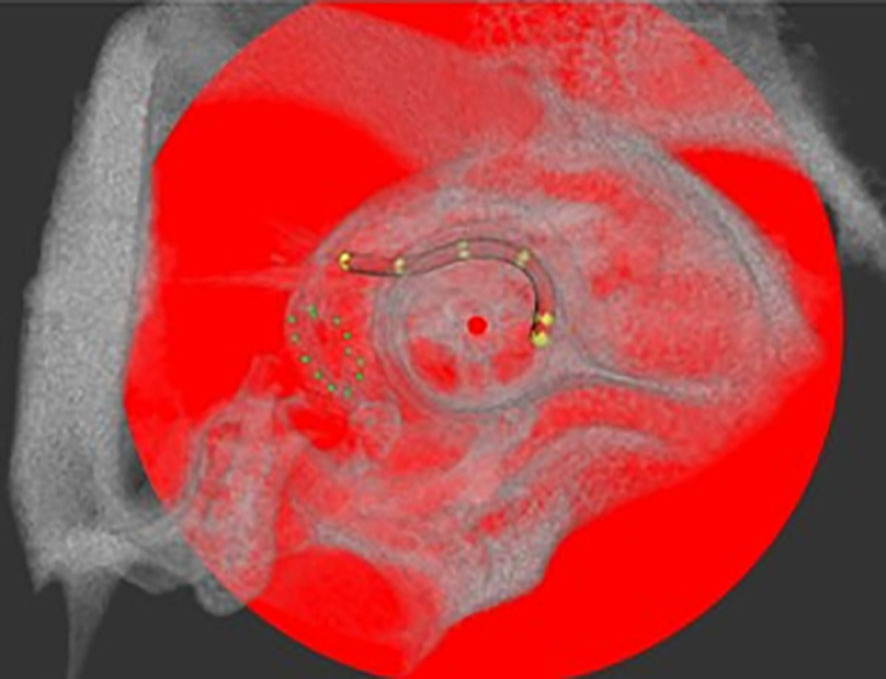
Example of electrode array position in the guinea pig cochlea after implantation via cochleostomy analyzed with COMET in a µCT scan. The round window niche is circled by green dots and electrode position is indicated by yellow marker points, set for implantation depth analysis. Center of the modulus as rotation axis is labeled with a red dot.

To detect differences in the implant-associated tissue reaction between animals, electrode position and degree of connective tissue growth in the implanted inner ears were assessed for the long-term implanted animals (GP004-011). To determine the implants’ position in the cochlea and possible movement over time, each guinea pig was scanned on day 0 immediately after implantation and at the end of the experiment using a µCT scanner (XtremeCTII, ScancoMedical AG, Switzerland). Scans were performed at 1,470 µA and 100 W at an integration time of 90 ms. The data were converted to DICOM, and the position of the electrode array was analyzed visually using the custom research tool “COMET” (25, 26). The program allows for the orientation of a slicing plane along the modiolar axis. The image plane can be rotated around this axis, thus always cutting the cochlear scalae radially for optimal segmentation conditions. The round window area was identified after positioning the rotation axis in the midmodiolar region. Starting from the middle of the cochleostomy on the cross-sectional contours of scala tympani, marker points were placed manually up to the tip of the electrode array (most apical contact) (**Figure 2**). The software directly reports the length of the insertion path in millimeters. A dislocation of an electrode would not lead to an exclusion from the analysis, since the focus of the study is not on the functional aspects of the electrodes after (re-)implantation, even though the literature suggests that the outcome depends on the positioning of the electrode (27–29).

Finally, all animals were euthanized for tissue harvesting to perform histology. To assess the tissue growth around the electrodes, temporal bones were removed during dissection and the implant was secured in place at the cochleostomy using Tetric EvoFlow^®^. The electrode was kept *in situ* to exclude loss of implant-surrounding connective tissue due to explantation. Temporal bones were opened to expose the cochlea and fixed with 4% PFA (paraformaldehyde) for 1 h on ice or overnight at 4°C. In the next step, tissue was decalcified for about 3 weeks in 10% ethylenediamine tetraacetic acid-disodium salt (EDTA, Sigma Aldrich Chemie GmbH, Germany) with periodic EDTA change. Decalcified tissue was trimmed to separate the cochlea. After dehydration with ethanol, the cochleae were cleared in Spalteholz solution [methyl salicylate and benzyl benzoate (MSBB)] and viewed on a Leica TCS SP8 confocal laser scanning microscope (CLSM) (30).

**Figure 3:**
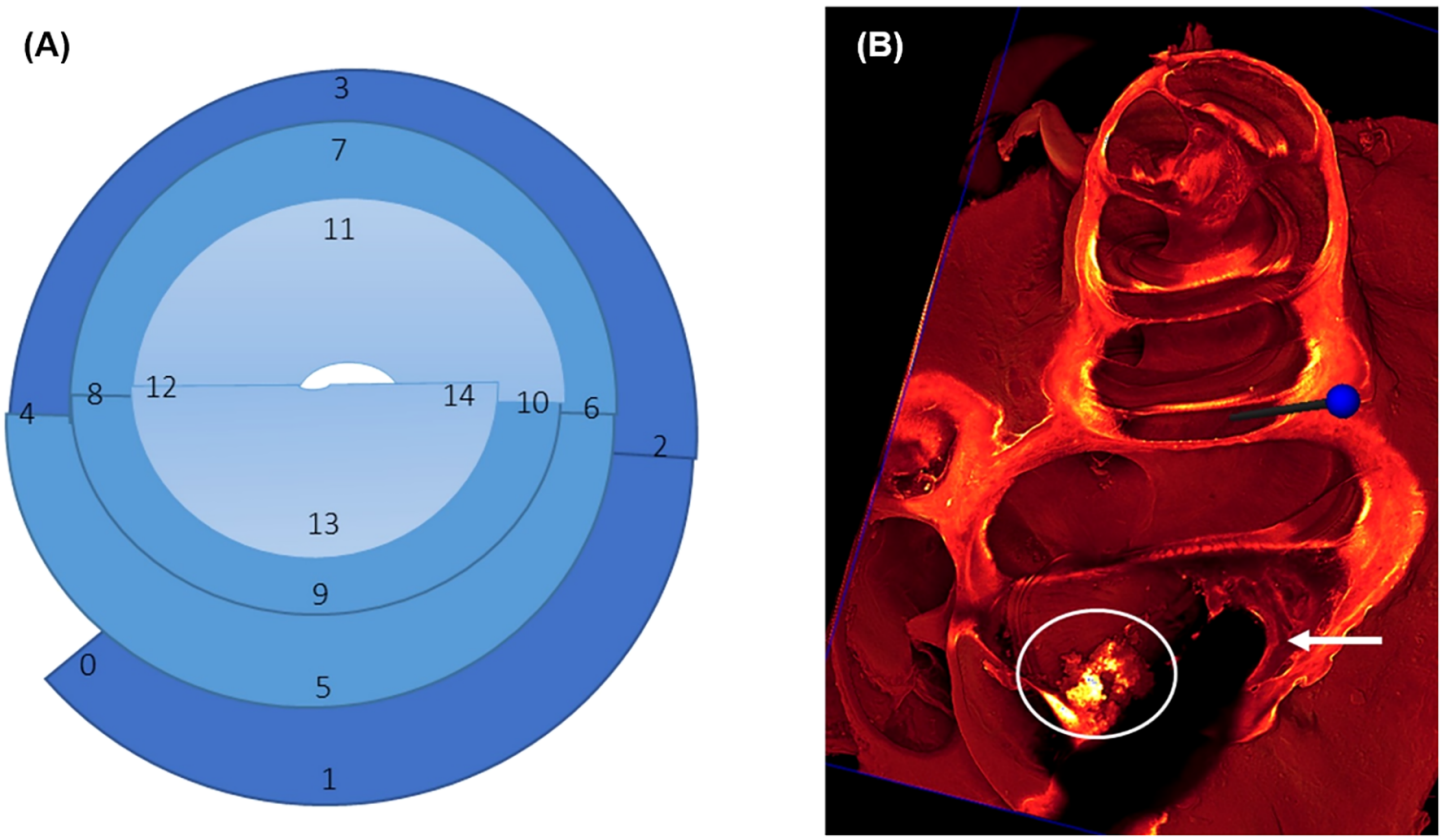
**(A)** Graphical illustration of regions of interest (ROIs) to be analyzed for tissue growth around the implant. The round window is marked as 0, 1 represents the region of cochleostomy, 1–4 cover the first turn of the cochlea corresponding to the basal region (dark blue), 5–10 cover the second turn with the middle region (middle blue), and 11–14 cover the third turn with the apical region (light blue). **(B)** shows an example of score 2 tissue growth with a thick cell layer (arrow) around the implanted electrode, which is visible as a dark shadow, while the scala is not filled completely with newly formed tissue (circle).

Tissue surrounding the implant is visualized due to its autofluorescence and evaluated using one representative image per region of interest (ROI). The scala tympani was divided into 14 ROIs (**Figure 3A**). Cleared cochleae were scanned with 10× objective (HC PL FLUOTAR 10×/0.30 DRY, Fa. Leica) and 20× objective (HC PL APO CS2 20×/0.75 IMM, Fa. Leica). A subjective evaluation of the tissue growth around the electrode was performed (e.g., **Figure 3B**) using a ranking system with the following scores: 0, no tissue (= free); 1, thin film of tissue, 1–2 cell layers (= low); 2, thick cell layers/scala not completely filled (= moderate); 3, investigated area of the scala tympani is (almost) filled with tissue (= high).

### Statistical analysis

Statistical analyses have been performed on the PET/CT measurements. The measured values for the cochleae (size in ccm, uptake of the radiopharmaceutical as SUVmean) were first tested for normal distribution using the Shapiro–Wilk test. In all cases, this test revealed a non-significant result (*p* > 0.05). Therefore, a normal distribution was assumed for all group comparisons. Accordingly, groups were compared using an unpaired *t*-test. Separately for [^18^F]FDG and [^68^Ga]FAPI-46 investigations, all non-implanted cochleae were pooled as a control group and then tested against the implanted cochleae. For [^18^F]FDG, implanted cochleae were either pooled as one group or split into groups studied early or late post-implantation. In the case of [^68^Ga]FAPI-46, only a group studied late post-implantation was compared to the controls. Additionally, paired *t*-tests were performed for a comparison of implanted and non-implanted cochleae in the respective animals. This was done for [^18^F]FDG early and late after implantation and for [^68^Ga]FAPI-46 at the late time point. An overview of the statistical results of this study is shown in **Table 3**.

**Table 3:** Overview of the results of statistical comparisons of different PET/CT parameters (cochlear volume and SUVmean) depending on the applied tracer ([^18^F]FDG and [^68^Ga]FAPI-46) and implantation status.

PET/CT parameter, group of cochlea with respect to tracer and implant status	Mean	Standard deviation
(a) Volume [ccm], FDG, non-implanted	0.016	0.002
(b) Volume [ccm], FDG early, implanted	0.015	0.002
(c) Volume [ccm], FDG late, implanted	0.014	0.001
(d) Volume [ccm], FAPI, non-implanted	0.016	0.002
(e) Volume [ccm], FAPI late, implanted	0.018	0.002
(f) SUVmean, FDG all, non-implanted	0.669	0.080
(g) SUVmean, FDG early, non-implanted	0.663	0.029
(h) SUVmean, FDG late, non-implanted	0.612	0.059
(i) SUVmean, FDG early, implanted	0.877	0.035
(j) SUVmean, FDG late, implanted	0.923	0.242
(k) SUVmean, FAPI all, non-implanted	0.535	0.112
(l) SUVmean, FAPI late, non-implanted	0.585	0.119
(m) SUVmean, FAPI late, implanted	0.656	0.020

While no significant differences were found between the compared cochleae with regard to volume, an increased [^18^F]FDG uptake was found in implanted cochleae examined early and an increased [^68^Ga]FAPI-46 uptake in implanted cochleae examined late after implantation compared to non-implanted cochleae. However, the small number of animals limits the explanatory power of the statistical tests; thus, results only give a preliminary tendency and have to be confirmed in more comprehensive studies. Results of statistical testing: a vs. b+c: p=0.2623, r^²^=0.0999; d vs. e: p=0.4837 r^²^=0.1892; **f vs. i: p=0.0006**, **r^²^=0.8207**; f vs. j: p=0.2743, r^²^=0.5067; **f vs. i+j: p=0.030**, **r^²^=0.5599**; **g vs. i: p=0.0416**, **r^²^=0.9186**; h vs. j: p=0.2785, r^²^=0.5205; **k vs. m: p=0.0393**, **r^²^=0.4788**; l vs. m: p=0.4272, r^²^=0.3281.

## Results

### PET/CT measurements

#### Cochlear volumes

**Figure 4:**
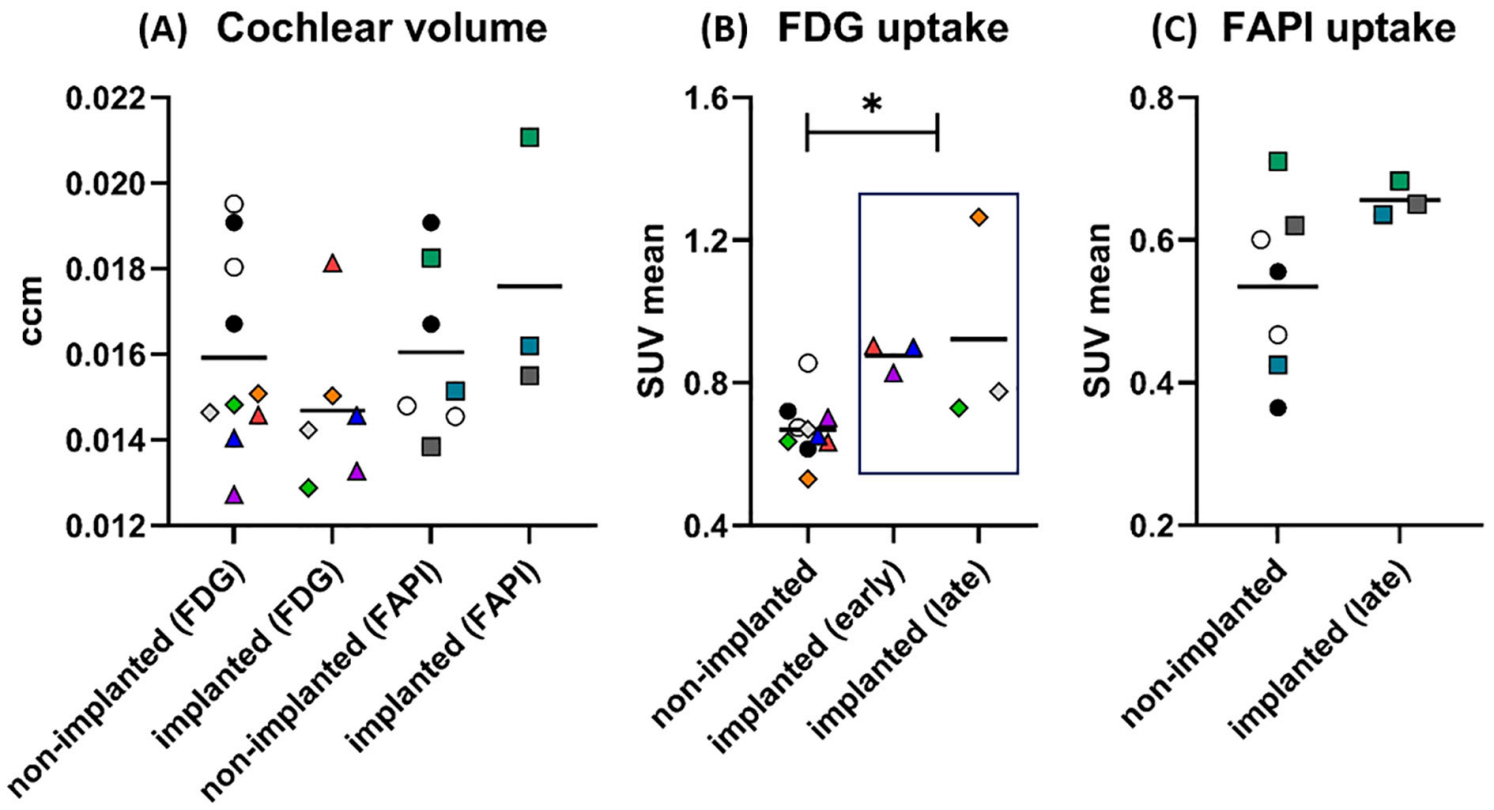
Results of PET/CT after cochlear implantation in guinea pigs. In graph **(A)**, the size of the cochleae (in ccm) measured by CT is shown separately for PET/CTs with [^18^F]FDG and [^68^Ga]FAPI-46. There are apparently no differences in size between implanted and non-implanted cochleae for either tracer. The data points represent the number of animals and cochleae. Non-implanted (FDG): *n* = 6 animals, *n* = 10 cochleae. Implanted (FDG): *n* = 4 animals, *n* = 6 cochleae. Non-implanted (FAPI): *n* = 5 animals, *n* = 7 cochleae. Implanted (FAPI): *n* = 3 animals and cochleae. For [^18^F]FDG, circles mark the control animal, which received no implants (black dot: GP001; white circle: GP002), triangles mark the animal imaged early after implantation (GP003: red: 7 days, blue: 14 days, purple: 21 days), and diamonds mark the animals imaged late after implantation (orange: GP004; gray: GP005; green: GP006). In case of [^68^Ga]FAPI-46, controls without an implant are likewise marked with a circle (black dot: GP001; white circle: GP002) and the implanted ones are marked with a square (green: GP008; dark gray: GP009; cyan: GP011). The symbols and colors have been assigned identically in graphs **(B, C)** The graph **(B)** shows the uptake (SUVmean) of [^18^F]FDG in the cochleae after implantation compared to the non-implanted cochleae. The uptake was significantly increased for the implanted cochleae pooled across time points (early and late) compared to all non-implanted cochleae. The data points represent the numbers of animals and cochleae. Non-implanted: *n* = 6 animals, *n* = 10 cochleae. Implanted (early): *n* = 1 animals, *n* = 3 cochleae. Implanted (late): *n* = 3 animals and cochleae. The graph **(C)** shows the results for the tracer [^68^Ga]FAPI-46. A long period after implantation (“late”), the mean uptake in the implanted cochleae was higher than the mean uptake in all non-implanted cochleae. The data points represent the numbers of animals and cochleae. Non-implanted: *n* = 5 animals, *n* = 7 cochleae. Implanted (late): *n* = 3 animals and cochleae. The horizontal lines in the graph always indicate the mean of the respective group. *p < 0.05. The numerical results of the statistical analysis are summarized in **Table 3**.

The guinea pig cochlear volumes were evaluated using the VOIs obtained from the low-dose CT scans. In the [^18^F]FDG PET/CT scans, there were no significant differences in the cochlear volumes between the implanted cochleae (0.0147 ± 0.0017 ccm) and the non-implanted cochlear controls (0.0159 ± 0.0022 ccm). In the [^68^Ga]FAPI-46 PET/CT scans, the cochlear volumes of the implanted cochleae (0.0176 ± 0.0025 ccm) did not significantly differ from the volumes of the non-implanted control cochleae (0.0161 ± 0.0019 ccm) (**Figure 4A**, **Table 3**).

#### [^18^F]FDG PET

**Figure 5:**
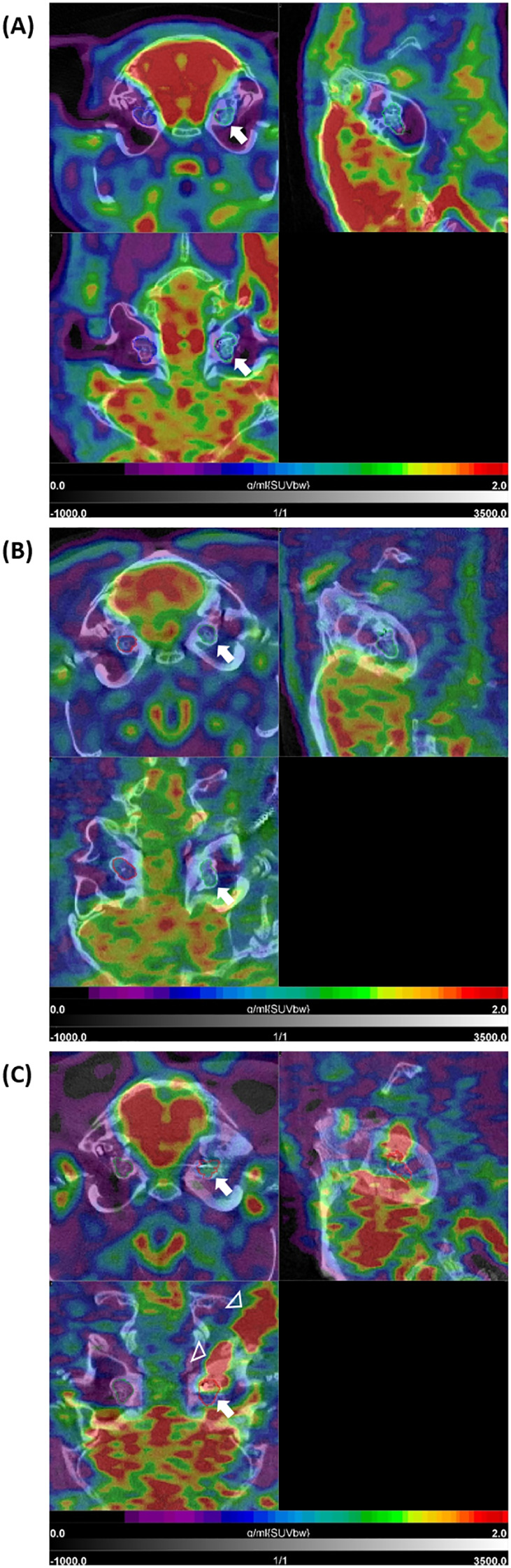
[^18^F]FDG PET/CT findings after cochlear implantation in guinea pigs. Shown are three different animals **(A–C)** with three orthogonal slices at the level of the cochlea. Very intense physiological uptake (red) is present in the brain (in all three examples). **(A)** shows moderately increased uptake in the area of the implanted left cochlea (white arrow) with intensity values in the blue-green sector of the color bar, compared to the non-implanted side (with purple-blue). In **(B)**, low uptake is present in the implanted left cochlea (white arrow) as well as the non-implanted right cochlea 1 year post-implantation. The example (GP004) in **(C)** shows clear increased uptake in the implanted left cochlea compared to the non-implanted right cochlea 1 year post-implantation (upper left tomogram, white arrow). This intense signal shows some local differences (heterogeneity) within the cochlea (lower left tomogram, white arrow) and is extended to the cable of the implant positioned in the retroauricular region (white arrowheads). SUVbw indicates that the standardized uptake value is normalized to the injected dose and body weight.

The described administration and detection via PET scan of [^18^F]FDG was successfully performed in the guinea pig model for cochlear implantation. Example images of different [^18^F]FDG signals in implanted and non-implanted cochleae are given in **Figure 5**. When pooling all time points, the [^18^F]FDG PET examinations show an increased SUVmean after implantation (0.900 ± 0.174) compared to the non-implanted controls (0.669 ± 0.080, *p* = 0.030). When splitting the implanted cochleae according to the two different time points, early (examinations at 1, 2, and 3 weeks in one animal) and late (examinations after 1 year in three animals) post-implantation, there was only a significant difference detectable between the controls and the cochleae of the animal examined early after implantation (0.877 ± 0.035, *p* = 0.0006) (**Figure 4**; **Table 3**). An example of increased [^18^F]FDG uptake early after cochlea implantation is shown in **Figure 5A**. The mean uptake obtained in [^18^F]FDG PET examinations late after cochlear implantation (SUVmean 0.923 ± 0.242) showed no significant increase compared to the uptake in control cochleae (*p* > 0.05). **Figure 5B** shows an example of bilaterally low [^18^F]FDG uptake in the cochleae 1 year after left-sided implantation, while in **Figure 5C**, a clearly increased unilateral [^18^F]FDG uptake is visible, underlining the heterogeneity at this time point. The SUVmean values of the controls and the implanted cochleae for the two examination time points are shown in **Figure 4B**. Additionally, an increased uptake was found when comparing the implanted cochleae (SUVmean 0.877 ± 0.035) to the non-implanted cochleae (SUVmean 0.663 ± 0.029) in the same animal early after implantation (*p* = 0.0416), but not when comparing the non-implanted cochleae with the implanted ones in the three animals late post-implantation (0.612 ± 0.059 vs. 0.923 ± 0.242; *p* = 0.2785).

#### [^68^Ga]FAPI-46 PET

**Figure 6:**
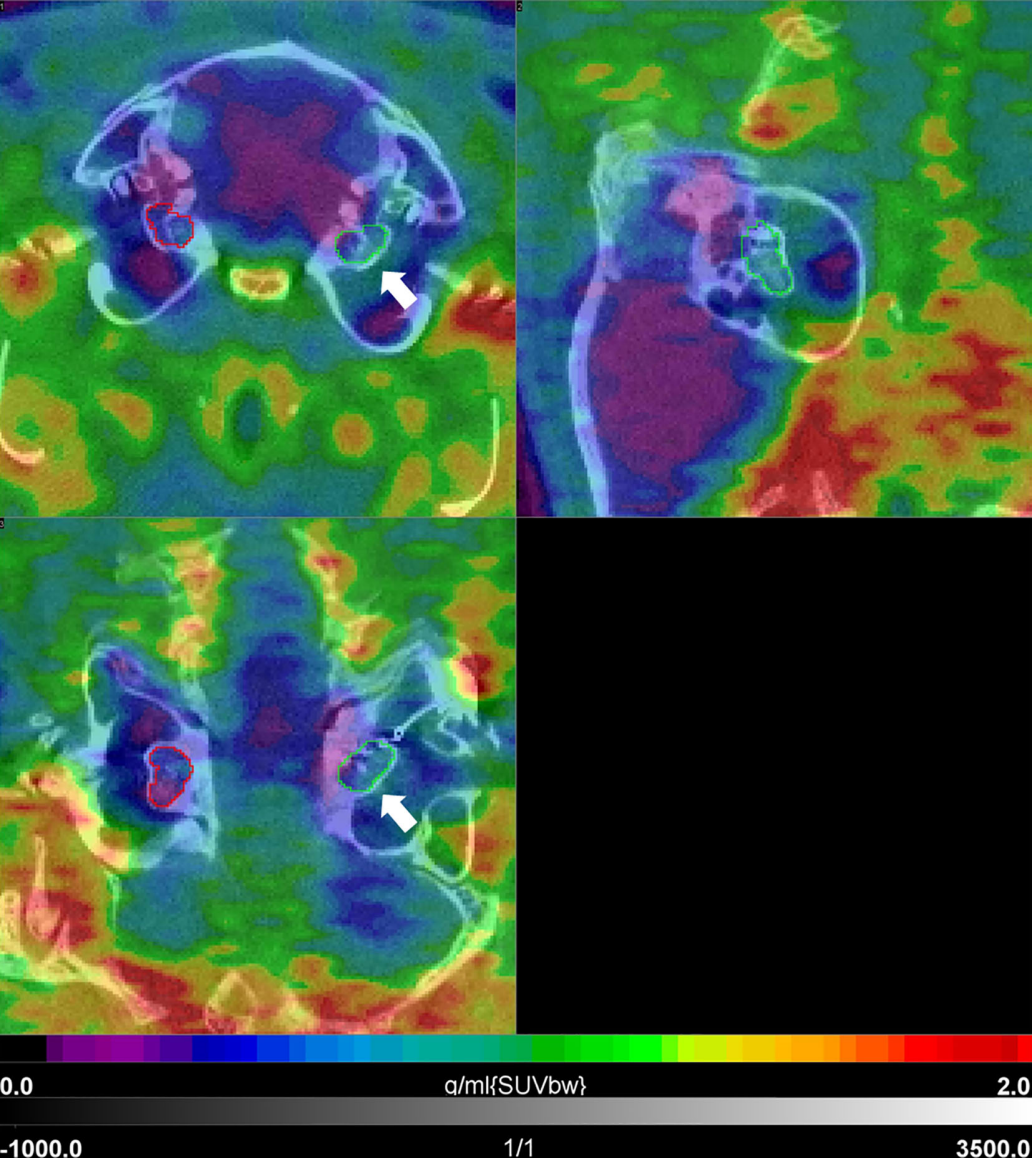
[^68^Ga]FAPI-46 PET/CT 1 year post-implantation in a guinea pig. Shown are three orthogonal slices at the level of the cochlea. There is a relatively slight increase in uptake in the area of the implanted left cochlea (white arrow) with intensity values in the blue-green sector of the color bar, when compared to the non-implanted side (purple-blue). SUVbw indicates that the standardized uptake value is normalized to the injected dose and body weight.

PET/CT of [^68^Ga]FAPI-46 was successfully used to assess fibrosis in the guinea pig model for CI implantation. [^68^Ga]FAPI-46 scans were only carried out late, 9–12 months post-implantation. Compared to [^18^F]FDG-imaging, SUVmean values were much lower in the implanted cochleae, albeit significantly higher in comparison to the pooled non-implanted control cochleae (0.656 ± 0.020 vs. 0.535 ± 0.112, *p* = 0.0393, **Figure 4C**; **Table 3**). A paired *t*-test revealed no significant difference in [^68^Ga]FAPI-46 uptake between the three cochleae of the implanted animals compared to their non-implanted cochleae (0.656 ± 0.020 vs. 0.585 ± 0.119, *p* = 0.4272). **Figure 6** shows an example of an animal examined with [^68^Ga]FAPI-46, with an increased uptake in the implanted cochlea in comparison to the non-implanted cochlea.

### Electrode position and histology

#### Insertion depth

Analysis of electrode insertion depth in µCT scans with COMET was successfully adapted to the guinea pig model. This enabled a simple and rapid way to measure the insertion depth and length in millimeters. In the performed experiments, the detected depths showed differences between animals and type of implanted electrode (**Table 4**). Depending on the electrode type, insertion depth directly after surgery ranged from approximately 2 mm (GP006) to 5 mm (GP005) for electrodes with five contacts and approximately 2.8 mm (GP008) to 3 mm (GP009) for electrodes with four contacts. A passive electrode with wire was only implanted in one animal, resulting in an insertion depth of 2.40 mm. Thus, overall, the deepest insertion was performed with electrodes with five contacts. Comparing the measured insertion depths directly after implantation with the ones measured at the end of the experiment (9 to 12 months), there is a slight increase (<0.10 mm) over time detectable in GP004 and GP011 and a larger increase (>0.10 mm) in GP005, GP008, and GP009. Only one animal (GP006) showed a decrease of 0.10 mm in measured insertion depth over time.

**Table 4:** Overview of implantation status, insertion depth, and tissue growth around the electrode for the individual animals.

Animal, implant	Implant status	Special findings	Insertion depth post-op [mm]	Insertion depth final (9 or 12 months post-op) [mm]	Movement [mm]	Degree of tissue growth (base to apex)				
						Basal 1	Basal 2	Basal 3	Basal 4	Mid 5
GP004, 5 contacts	5 contacts in cochlea	4. Contact dislocated into scala vestibuli; atrophy of the retroauricular tissue adjacent to the electrode	3.82	3.84 (9)	+0.02	2	2	2	1	0
GP005, 5 contacts	5 contacts in cochlea	3. Contact loosened and pressing on lateral wall	5.16	5.31 (12)	+0.15	1	2	3	3	3
GP006, 5 contacts	2 contacts in cochlea		2.11	2.01 (12)	-0.10	2	3	3	0	0

GP008, 4 contacts	3 contacts in cochlea		2.77	3.02 (12)	+0.25	2	3	1	0	0
GP009, 4 contacts	4 contacts in cochlea		2.95	3.41 (9)	+0.46	2	3	1	0	0
GP011, passive, with wire	3 contacts in cochlea		2.40	2.48 (9)	+0.08	2	2	0	0	0

A decrease in insertion depth over time is labeled in gray, a slight increase is labeled in light blue, and a noticeable increase is labeled in dark blue. Tissue growth is scored as follows: 0 = free (green); 1 = low, 1–2 cell layers (light brown); 2 = moderate, thick cell layer, scala not completely filled (middle brown); 3 = high, scala completely filled (dark brown). The horizontal double line separates the animals analyzed with FDG (upper three) or FAPI (lower three).

#### Degree of tissue growth

Animals were implanted via cochleostomy, and it is notable that no animal showed fibrosis in the round window area, but bone dust due to the drilling of the cochleostomy was detectable at the basal side of the cochleostomy in GP005. During temporal bone preparation at the end of the experiments (9–12 months), we observed that in the implanted side, the bullae were often filled with connective tissue and both the bulla and cochlea were intensively ossified. Additionally, GP004 had a dislocation of the fourth contact into the scala vestibuli and an atrophy of the retroauricular tissue covering the extra-bullar part of the electrode. In GP005, the third contact of the electrode was loosened and pressed against the lateral wall. The degree of tissue growth around the electrode was analyzed in confocal scans of the cleared cochleae and results for the animals are summarized in **Table 4**. The tissue growth around the implant was limited in all animals to the basal part of the cochlea in the area of electrode insertion and did not reach apically beyond the electrode tip to the middle region of the cochlea. The only exception was GP006, where tissue growth exceeded the electrode tip to the first middle region. In two animals (GP004 and GP011), tissue growth was limited to a moderate degree, while all other animals had a high degree of tissue growth in at least one cochlear region.

Combining the results of detected change in electrode insertion depth with the assessed amount of tissue growth, there is a tendency for more tissue around the implanted electrodes with more variations over time (**Table 4**). Only animals with a change ≥0.10 mm showed a high degree (3) of tissue growth.

#### Animal-specific correlation of insertion depth and PET intensity with intracochlear tissue growth

A statistical comparison of the results obtained with the different methods in this pilot study is not possible due to the low number of animals per group. Therefore, comparisons have to be based on individual findings and give only a first hint for interpretation.

**Figure 7:**
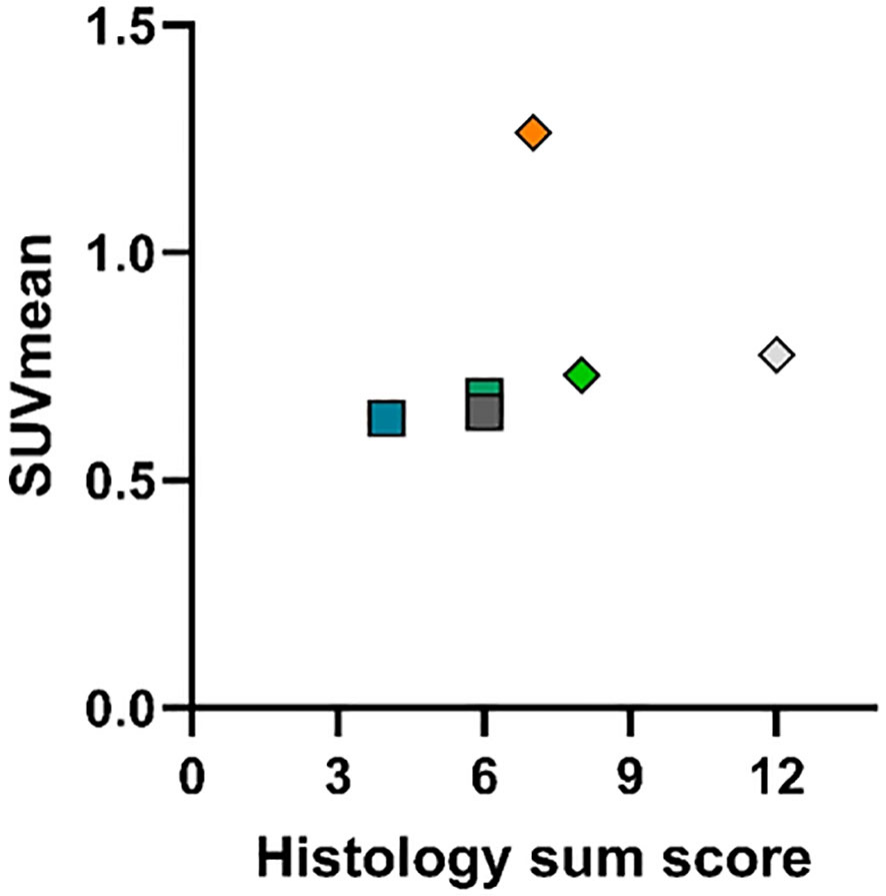
Comparison plot of summed tissue growth score vs. SUVmean in [^18^F]FDG and [^68^Ga]FAPI-46 imaging late post-implantation. For the assignment of symbols to the respective animals, see the caption of **Figure 4**. Histology sum score expresses the summation of all regional tissue growth scores in the corresponding cochlear. For SUVmean values, see **Table 3**, and for tissue growth scores, see **Table 4**. For both tracers, no correlation between uptake and tissue growth is obvious in the comparison plot.

In total, there was no obvious correlation between implanted electrode type, insertion depths, and extent and intensity of tissue growth (see **Table 4**). GP006 received a five-contact electrode and showed the highest amount of tissue around the implant, most intense in region basal 3 to mid 5 along the scala tympani, and had the deepest insertion of the electrode of all animals. Comparatively, GP004, which was implanted with the same electrode and at a similar depth, showed only a low (basal 4) to moderate (basal 1–3) tissue growth around the electrode. The last animal implanted with a five-contact electrode (GP006) had the shortest insertion depth but a moderate (basal 1) to high (basal 2–3) degree of tissue growth. The two animals with the four-contact electrodes had a medium deep insertion (within analyzed animals), and tissue growth ranged from moderate (basal 1) to high (basal 2) to low (basal 3). GP011 was implanted with a passive electrode, which was not inserted deep, and a moderate amount of tissue growth extended only to the basal 2 region. **Figure 7** compares the results of tissue growth with the PET intensity.

Combining the results of the PET scans with the results for tissue growth, there is no obvious correlation (**Figure 7**). In the FDG studies, exceptionally increased activity was detectable in one animal (GP004) 1 year after implantation, but for the total group of animals examined after this interval, no significant increase was detected.

## Discussion

To date, post-operative observation of the cochlear health of patients receiving a CI is limited to electrophysiological and audiological measurements and radiological imaging (7, 31–34). An assessment of, e.g., the tissue status after cochlear implantation is currently only possible through histological analyses post mortem. Because of the lack of a non-invasive diagnostic tool, it is not possible to get a deeper understanding of how ongoing tissue reactions in the implanted inner ear are associated with differences in CI performance or loss of residual hearing. This deficiency additionally hinders progress in targeted therapeutic interventions and follow-up examinations. Animal studies allow for investigation of some of these questions; however, they are very limited in their ability to assess CI performance (e.g., speech recognition testing), and histology and molecular biological investigations are time-consuming and expensive (35). Furthermore, these methods do not allow longitudinal observation post-implantation in the same animal. Groups of animals must be observed and sacrificed for the different examination time points, resulting in a large number of animals required per experiment. Nuclear medicine methods could be a solution for this problem, but it must be shown whether application and detection in the cochlea is possible and whether implant-associated changes or change in cochlear health can be detected. There are a number of established and new PET biomarkers for inflammatory processes and fibroblast activation. In particular, these methods have already proven their suitability for detecting macrophage activation in small structures (e.g., inflamed large vessel walls) or a foreign body reaction (8–10, 36, 37). Likewise, radioactively labeled molecules that bind to activated fibroblasts (so-called FAPI tracers) have proven useful for the evaluation of molecular changes after medical interventions—especially those involving foreign body response after implantation (38).

The aim of this pilot study was to investigate if detection of PET radiotracers in cochlea-implanted guinea pigs is feasible and whether results correspond to histological findings of tissue growth around the implants. To choose the most promising radiopharmaceuticals for this pilot study, pathophysiological changes after cochlear implantation known from animal experiments and in post-mortem examinations of CI patients (39–44) were used as targets. The experimental studies showed that there is usually an inflammatory/foreign body and a fibrotic reaction around the implant to varying degrees (39, 40). The inflammation is characterized by a recruitment of leukocytes into the cochlea as well as an activation of resident macrophages (40). The foreign body reaction is associated with the formation of multinuclear foreign body giant cells (41). Furthermore, there is also an activation of fibroblasts and, as a consequence of all these processes, there can be damage to the spiral ganglion neurons (40, 41, 43). Fibrosis can occur in the tympanal scale with encapsulation of the implant, which impairs the transmission of signals from the electrode to the nerve and damages the architecture of the cochlea (44, 45). With this background, [^18^F]FDG was chosen as the PET tracer to detect inflammation, which is characterized by an increase in glucose metabolism leading to an accumulation of the radioactively labeled glucose analogue. Fibrosis around the implant in the later phase post-implantation could be visualized with [^68^Ga]FAPI-46, as it binds to activated fibroblasts (38). These approaches were tested in a pilot study using a guinea pig model with EIT for the CI and high-resolution preclinical PET imaging.

Initially in this pilot study, imaging of the cochlea was established in non-implanted animals using different radiopharmaceuticals in separate PET acquisitions in the same animal to show that the mean SUV in the cochlea can be reliably determined as a biomarker of physiology. The examination with different radiopharmaceuticals in the time course was also feasible. With the radiotracer [^18^F]FDG, it was then possible to detect inflammatory changes over time. Directly after implantation, it is expected that there is an acute phase of inflammation with metabolically very active cells (indicated by high FDG uptake), followed by a chronic phase with lower cell activity in the fibrotic/bony sheath (indicated by lower FDG uptake). This was the case in our study. There was a decrease in FDG intensity detected between one animal scanned three times within the first three weeks after implantation (GP003) and the animals scanned 1 year post-implantation (GP005, GP006; GP004 was rated as a outlier due to implant translocation and a potential contamination of the FDG signal from outside the cochlea). It was also possible to demonstrate a generally increased fibrotic activity at the late stage with the use of [^68^Ga]FAPI-46. Thus, both pathophysiologic processes could be detected at a molecular level and visualized in a model for traumatic electrode insertion in guinea pigs. In particular, the temporal sequence with an early phase dominated by the inflammatory response and a later phase with increasing fibrotic tissue formation fits the experimental results and hypotheses on the pathophysiology of molecular changes post-implantation (40, 43, 46).

After the final PET/CT scan, the cochleae of the six animals implanted 9–12 months before were examined histologically to compare PET results with the degree of tissue growth around the implant in the individual animals. The performed EIT successfully induced tissue growth in all implanted cochleae, which was not obviously dependent on the implanted electrode type and insertion depth in this small experimental group. Except for two animals with a maximally moderate degree of fibrosis (GP004 and GP011), all other animals showed at least, in one of the analyzed regions of the cochlea, a high degree of tissue filling of the scala tympani. Interestingly, there was a tendency for more tissue growth around electrodes that had a higher change (≥0.1 mm) in measured insertion depth over time. These changes in insertion depths can be due to small deviations in setting of the measuring points in COMET but could also indicate different degrees of electrode movement during the observation time. Electrode migration is reported to be often accompanied by implant-associated fibrosis or ossification, whereby it is discussed that the tissue growth may cause the extrusion of the implant (47). Our study included cutting the connection to the connector or implantation of a short dummy electrode and detected mainly an increase in electrode depth over time. Because of the missing connection, there is no restraining function as is the case in CI patients with an intact connection to the receiver. Such a connection could have prevented the (apical) movement seen here ranging up to 0.46 mm (GP009). It would be plausible that the underlying inward and outward forces during movement have induced the here seen increase in electrode encapsulation.

When considering the individual results of our pilot study, no clear correlation could be found between the degree of tissue growth around the implant and the intensity of inflammation (FDG) or fibroblast activation (FAPI) in the individual animals (**Figure 7**). One reason for this could be that tissue growth in the scala tympani was assessed in sections, while the PET scan signal was averaged over the whole cochlear region. Another limitation of this pilot study is that only the quantity of tissue around the implanted electrode was assessed, not the cellular composition of the tissue. In this respect, PET could potentially provide additional information, since high glucose consumption/FDG uptake and low fibroblast activation/low FAPI uptake primarily indicate active inflammation, whereas relatively lower FDG uptake with high fibroblast activation/high FAPI uptake indicates a rather chronic inflammatory environment with ongoing fibrosis. This is in good agreement with the results of the present pilot study. An increased glucose uptake (FDG uptake) was observed as a sign of expected acute inflammation (4) early on (a few weeks) after implantation but not later (after 9–12 months). However, we have not yet provided evidence of the expected low fibrotic activity early on in this pilot study. At a late stage after implantation, however, we did detect the expected significant increase in fibrotic activity based on increased FAPI uptake—without significantly increased FDG uptake, i.e., without inflammatory activity.

However, a different constellation of findings emerged in the following case in which special additional factors probably played a role. Animal GP004 had a comparatively exceptionally high uptake of FDG in the PET scan, indicating a very active glucose metabolism in the cochlea, which is typical for a strong inflammation, while the detected tissue growth in the scala tympani was only moderate. In the histological assessment of this animal, we noted a translocation of the electrode into the scala vestibuli. The massive trauma underlying this translocation could be the reason for the intense, chronic inflammation up to 1 year post-implantation. A translation of the PET methodology to the patient as a diagnostic tool could help to identify possible reasons for a poor outcome post-implantation. Fibranz et al. showed that in case of a dislocated electrode in a guinea pig, the tissue growth was mainly located in the scala vestibuli, which was not analyzed in our study, but could of course contribute to a higher FDG-PET signal in the whole cochlea (48). Additionally, in GP004, there was a conspicuous tissue reaction in the retroauricular region around the cut end of the electrode and reaching up to the cochlea. It was visible macroscopically by local tissue remodeling (without any other abnormalities shown by the animal), and a very high activity in the FDG-PET scan indicated local inflammation. This co-localized inflammation might have additionally triggered the tissue and FDG response in the cochlea. Finally, the high FDG signal measured for the cochlea might partly be a consequence of the imaging methodology. It is well known that in PET images, areas with high activity concentration neighboring an area of interest can result in an overestimation of the activity in the area of interest—the so-called spillover effect (49). Therefore, it cannot be excluded in the present case that the high signal in the cochlea is partly driven by the high signal of the local inflammation next to the cochlea.

The [^68^Ga]FAPI-46 PET signal was significantly elevated in implanted cochleae with little variation. In the histological evaluation, two of these animals (GP008 and GP009) were scored with the same high tissue growth, and one (GP011) was scored as moderate. This suggests that there are activated fibroblasts in the tissue surrounding the implant. However, the higher scores of GP008 compared to that of GP009 are not reflected in the considerably higher FAPI binding. Cell-specific staining might elucidate a potential correlation between *in vivo* and *in vitro* measurements of fibroblast activation, but it was not included in this pilot study.

Nevertheless, our pilot study was able to establish and prove the feasibility of high-resolution PET imaging in the cochlear implanted guinea pig model with [^18^F]FDG and [^68^Ga]FAPI-46. The results presented here encourage further investigation of this method to finally establish in the clinic a non-invasive *in vivo* measuring tool to detect molecular changes in the cochlea. There is already extensive experience with the use of PET biomarkers, in terms of both their safety and their suitability for detecting pathophysiological mechanisms that are also involved in CI treatment. Additionally, the resolution of the current PET device generation is so high (transaxial full width at half maximum of approximately 3.3 mm) that it is sufficient for the signal detection of approximately 60% of true activity from the human cochlea with average dimensions of 5.14 mm (2.8–6.9 mm) in height and 6.4 mm (6.22–6.86 mm) in basal width (21, 50, 51). At the same time, the arsenal of possible preventive/therapeutic measures to prevent pathophysiological changes associated with CI is increasing. Both of these developments should benefit from each other. The molecular processes detectable with PET biomarkers would allow the evaluation of a wide range of anti-inflammatory and anti-fibrotic therapeutic approaches. These include intracochlear application of pharmaceuticals, implantation that is as atraumatic as possible or the use of materials that promote this, and the implantation of electrodes that release pharmaceuticals (43, 44, 52, 53). This also appears desirable in view of the fact that these changes represent a significant factor for variable speech comprehension after CI implantation and in view of the increasing availability of pharmacological therapies (33, 45). Local therapies could be examined *in vivo* in patients for the first time using molecular imaging, which could be key to implementing effective therapies.

Indications like device failure, infections, extrusion of the implant, and cholesteatoma could necessitate the reimplantation of a CI (54). It has been shown that, during reimplantation, the new electrode follows the same path in the scala, and cochlear coverage seems to be limited by this (55). Speech recognition tests after reimplantation due to a device failure brought little to no improvement (56, 57). Reasons for the lack of improvement in performance after reimplantation of an even technically upgraded implant are not clearly identified yet, but intracochlear fibrosis and inflammation are assumed to be involved (55). The establishment of PET imaging as a tool to evaluate inflammatory and fibrotic processes inside the cochlea could provide insight into the involved mechanisms and possibly enable an assessment of the success of this procedure. However, further studies (preclinical and clinical) are necessary to reliably evaluate or even predict the reimplantation outcome on the basis of PET imaging results. Once established, this can be done before and/or after the reimplantation, in the latter particularly to observe the effectiveness of additive pharmacological treatments.

Moreover, the diagnostic potential of visualizing molecular processes in the cochlea goes beyond the context of implantation. In this way, other pathological processes, particularly neurodegenerative processes in the cochlea, could be detected without intervention as well. This could be particularly interesting in the context of the current discussion about a link between hearing loss on the one hand, and cognitive decline on the other. Hearing loss and dementia show common radiological and biological findings, although this does not imply a clear cause-and-effect relationship (58). This is also reflected in the following observations: on the one hand, animal data show that hearing disorders can lead to dysfunction of cerebral cortical areas such as the hippocampus, which, in turn, causes cognitive impairment (59). On the other hand, different forms of dementia (e.g., Alzheimer’s disease or dementia with Lewy bodies) lead to different phenotypes of hearing impairment (60). One possible direct pathomechanism of the manifestation of hearing impairment in dementia would be an involvement of the cochlea. To date, Alzheimer’s-related pathology has been observed in the retina, but is not established for the cochlea (61). At least in animal experiments, a deposition of tau protein in the cochlea could be demonstrated in a transgenic Alzheimer’s mouse model (62). Therefore, further molecular investigations of neurodegenerative markers in the cochlea in patients with cognitive decline would be of interest in light of the unanswered questions regarding the link between hearing loss and dementia.

## Conclusion

This pilot study showed that preclinical PET/CT can be used to detect molecular processes in the guinea pig cochlea. A few weeks after traumatic electrode insertion, the radiopharmaceutical [^18^F]FDG showed increased activity in the cochlea, indicating an inflammatory reaction. [^68^Ga]FAPI-46 showed increased fibroblast activation 1 year post-implantation. A clear correlation between PET signal and the amount of tissue growth around the electrode could not be detected in this pilot study, although the demonstrated presence of tissue growth is in line with molecular processes illustrated by PET imaging. Future, larger studies, which combine PET imaging methods established here with cell-specific histology and biomolecular detection of pro-inflammatory and fibrotic markers and measurement of gene expression, may be able to prove such correlations. This would enable the use of PET/CT for *in vivo* follow-up in the development of new anti-inflammatory or anti-fibrotic therapeutic approaches post-implantation. Furthermore, it could pave the way for these new approaches to be used in clinical practice, thereby benefiting CI patients both by the increased understanding of underlying pathophysiological mechanisms and by identifying possible novel implantation-related therapies. In addition, possibilities open up to characterize interdependencies between hearing loss and dementia in more detail.

## Data Availability

The raw data supporting the conclusions of this article will be made available by the authors, without undue reservation.
